# Nursing Interventions for Post-Intensive Care Syndrome in Follow-Up Clinics: A Scoping Review

**DOI:** 10.3390/nursrep16020055

**Published:** 2026-02-05

**Authors:** Telma Gonçalves, Marta Santos, Patrícia Pontífice-Sousa, Vanessa Antunes, Lúcia Bacalhau

**Affiliations:** 1 Escola de Enfermagem de Lisboa, Universidade Católica Portuguesa, 1649-023 Lisbon, Portugal; telma.vieira.goncalves@hospitaldecascais.pt (T.G.);; 2 Center for Interdisciplinary Research in Health, Universidade Católica Portuguesa, 1649-023 Lisbon, Portugal; 3 Egas Moniz Center for Interdisciplinary Research (CiiEM), Egas Moniz School of Health and Science, 2829-511 Almada, Portugal

**Keywords:** post-intensive care syndrome, nursing interventions, follow-up, intensive care units, critically ill patients, family

## Abstract

The rise in ICU survival rates has introduced new challenges related to the long-term effects of intensive care, known as Post-Intensive Care Syndrome (PICS). Nurses play a key role in these clinics; however, the nature and outcomes of their interventions remain insufficiently understood. **Objectives**: This review aims to identify nursing interventions for PICS in follow-up clinics. **Methods**: Using the JBI scoping review methodology, we searched PubMed, Web of Science and CINAHL (via EBSCOhost) in March 2025, and examined grey literature in RCAAP and Open Dissertations (through B-ON). Inclusion criteria, based on JBI’s PCC (Population, Concept, Context), focused on nursing interventions for PICS for patients and families in follow-up. Studies involving children, adolescents, pregnant women, and those receiving end-of-life care were excluded. **Results**: Encompassing studies from 2005 to 2022 across multiple countries, this review highlights nursing interventions for post-ICU recovery. A total of 394 articles that met our search criteria were found, resulting from searches in the mentioned databases. These were initially exported to Rayyan, and 115 duplicates were removed. The 21 articles that met our inclusion criteria were fully analyzed, and those that effectively answered our questions and met our inclusion criteria were selected. In the end, 9 articles were selected, to which, after an individual analysis of their bibliographic references, 3 more were added, totaling 12 articles submitted to the final analysis. **Conclusions**: For patients, interventions ranged from debriefing, PICS symptom evaluation, ICU re-visits, health education, cognitive–behavioral therapy and support groups, complemented by home-based physical rehabilitation and virtual reality. Family-focused interventions centered on appointment involvement, educational sessions, patient diary review, and emotional support. These assessments and interventions address the consequences of ICU admission, with the goal of facilitating physical, mental, and emotional rehabilitation of ICU survivors. This review emphasizes the critical role of follow-up consultations in the recovery of both patients and families. A comprehensive assessment using PICS scales and the integration of families into care plans are crucial for optimizing intervention outcomes. **Implications for Clinical Practice**: The development of evidence-based guidelines for implementation of follow-up clinics for SPICI appointments is necessary.

## 1. Introduction

In recent years, exponential advances in science and technology have brought remarkable changes to health and human longevity. However, with the increase in survival rates, new challenges arise, mainly concerning the long-term consequences of intensive treatments.

One of these challenges is post-intensive care syndrome (PICS), which affects between 25% to 50% of critically ill patients [1]. PICS is a syndrome composed of physical, cognitive and psychological symptoms that have a significant impact on the quality of life not only of patients after discharge but also of their families. Mental, physical, and cognitive health are affected, leading to the development of depression, anxiety, dementia, post-traumatic stress, respiratory pathologies, muscle weakness, and generalized pains. There are some risk factors for PICS, including prolonged ICU stay, mechanical ventilation duration, sepsis, delirium, and pre-existing frailty [2].

To address the complex challenges associated with post-intensive care recovery, follow-up clinics have emerged to monitor and support the rehabilitation of patients discharged from the ICU, as well as their families. These clinics operate through a multidisciplinary approach, integrating professionals from nursing, medicine, physiotherapy, psychology, occupational therapy, and speech therapy [3]. Nurses play a crucial role here, identifying early signs and symptoms of PICS and ensuring the patient’s transition to life outside the hospital as safely and effectively as possible [4].

Despite their central involvement, the nature and effectiveness of nursing interventions in this context remain insufficiently explored in the literature. This gap highlights the need to better understand how nurses contribute to the care of individuals living with PICS and their families within follow-up clinics.

Given the relevance of this topic and the growing recognition that structured post-ICU follow-up—which includes follow-up consultations as dedicated assessment moments within follow-up clinics—is a key component of patient recovery, it becomes essential to synthesize the available scientific evidence on nursing interventions implemented in this context. Therefore, this scoping review aims to map and describe the existing evidence related to nursing interventions for PICS delivered in follow-up clinics and their follow-up consultations, providing an overview of current practices and identifying gaps that can inform future research and clinical development.

## 2. Materials and Methods

This scoping review was conducted in accordance with the Joanna Briggs Institute (JBI) methodology [5], which provides a structured approach for mapping existing evidence on a given topic, and it was guided by the following research question: What are the nursing interventions (Concept) that aim to address PICS-related needs in adult patients and their families (Population) within follow-up clinics after ICU discharge (Context)?

The aim is to map the state of knowledge, identify gaps, and clarify concepts, especially in this area where evidence is not yet well established.

### 2.1. Protocol and Registration

This research was conducted in March 2025 according to a protocol written a priori and registered on the OSF platform on 14 October 2024, identified as ‘Nursing Interventions in Post-Intensive Care Syndrome in Follow-Up Clinics: Scoping Review Protocol,’ with the DOI: https://doi.org/10.17605/OSF.IO/KH5DT.

### 2.2. Eligibility Criteria

The eligibility criteria were defined using the PCC mnemonic: Population, Concept, and Context, as follows:

Population: Adults diagnosed with Post-Intensive Care Syndrome (PICS) and referred to follow-up clinics, as well as their families. Studies focusing on children, adolescents, pregnant individuals, or end-of-life patients were excluded.

Concept: Nursing interventions aimed at addressing PICS-related needs in patients and their families.

Context: Follow-up clinics or follow-up consultations conducted after discharge from the Intensive Care Unit (ICU).

As to the type of methodology, to amplify the coverage of scientific evidence, quantitative, qualitative, mixed, primary, and secondary studies that answer our research question were considered. Studies in Portuguese, English, Spanish, and Italian languages were included without temporal or geographical restrictions.

### 2.3. Search Strategy

An initial exploratory search was performed in the Open Science Framework, JBI Evidence Synthesis, and PubMed to identify any existing scoping reviews or protocols related to the topic. This search revealed no published or ongoing reviews addressing nursing interventions for PICS in follow-up clinics. Based on this, a comprehensive search strategy was subsequently developed for the main databases included in this review.

### 2.4. Information Sources

According to the PRISMA recommendations for conducting scoping reviews, the research strategy took place in three phases.

Initially, a preliminary search was conducted in Open Science Framework, JBI Evidence Synthesis, and PubMed to identify keywords and search terms and to verify the feasibility of the topic.

In the second stage, we constructed a search strategy based on the search terms and keywords for each of the selected databases: PubMed, Web of Science, and CINAHL through the EBSCO-HOST platform. In this stage, gray literature was also verified in the Open Access Scientific Repository of Portugal (RCAAP) and Open Dissertations through B-ON.

In the third and final stage, an analysis of the bibliographic references of the selected studies was performed to ensure that relevant articles to our research, which may not have been retrieved, were not excluded. A complementary search was conducted on reference websites for the topic, such as the European Society of Intensive Care and the Society of Critical Care Medicine.

### 2.5. Search

The electronic search strategy used a combination of search terms applied without field restrictions. The grouping and combination of terms were adapted to the structure and specific requirements of each platform and database. Full search strategy is available online at OSF https://osf.io/kh5dt/overview (accessed on 12 November 2025) and Table 1.

### 2.6. Selection of Sources of Evidence

**Figure 1:**
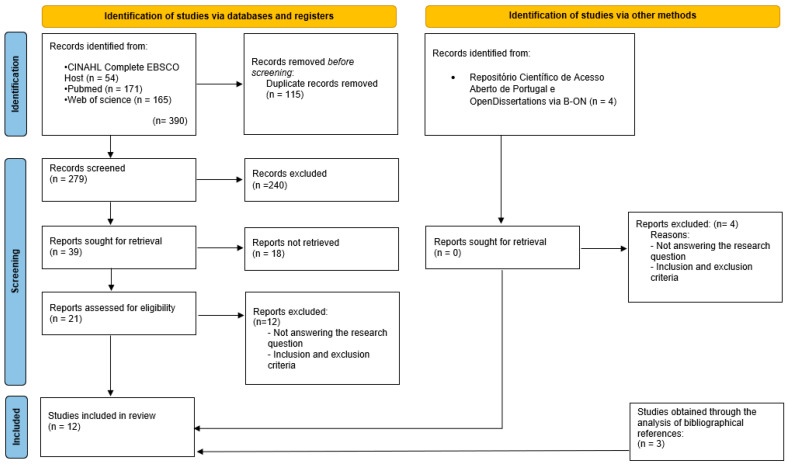
PRISMA Flow Diagram [6].

We identified 394 records. After removing 115 duplicates, 279 titles/abstracts were screened. Thirty-nine records were selected for full-text assessment (18 conflicts resolved by consensus). Twenty-one full texts were assessed for eligibility, and 9 studies met inclusion criteria. Reference checking yielded 3 additional studies, resulting in 12 included studies (Figure 1).

### 2.7. Data Charting Process

After selecting the articles included in the scoping review, data extraction was conducted independently by the two reviewers. The extracted information, focused on nursing interventions and their reported outcomes, was entered into a data extraction table designed to summarize and organize the evidence relevant to the objectives of the review. The initial extractions were then compared, and any discrepancies were discussed and resolved by consensus, ensuring accuracy and consistency in the charting process. Operational criteria were defined to consistently identify nursing-related interventions. Studies were included when interventions were led by nurses, co-led with a clearly identifiable nursing component, or integrated into multidisciplinary programmes with a clearly described nursing contribution. Multidisciplinary studies were categorised according to the explicit description of nursing roles or activities within the intervention.

### 2.8. Data Items

Table 2, based on the JBI recommendations (template study details, characteristics, and results extraction instrument) and adapted by us, summarizes the bibliographic characteristics, study type, population, objectives, nursing interventions, results of those interventions, and key points related to our research.

### 2.9. Synthesis of Results

After full reading of the included articles, the extracted data were organized into a main table constructed and validated by all authors. This table presents the general characteristics of the studies, as described in the data items section. Given that the primary aim of this review was to map the evidence on nursing interventions addressing PICS-related needs in follow-up clinics, and considering the diversity of interventions and reported outcomes identified across the studies, an additional table (Table 3) was developed to summarize the specific nursing interventions and the corresponding outcomes.

## 3. Results

Our focus is on analyzing nursing interventions, categorized into patient-focused and family-focused interventions, summarized in the outcomes presented in Table 2. Among the reviewed articles, not all addressed both types of interventions: three studies presented interventions for both patients and families, eight focused exclusively on patients, and one addressed family-specific interventions.

### 3.1. Study Characteristics

As detailed in Table 2, the included articles span from 2005 to 2022, originating from various countries including England, Scotland, Sweden, Netherlands, Iceland, Denmark, Norway, and the USA. All articles were published in English. The study designs comprised seven randomized controlled trials, one quasi-experimental prospective study, one ethnographic study, one comparative descriptive study, one systematic review and meta-analysis, and one cohort study.

The organization, professionals involved, consultation frequency, patient follow-up duration, and interventions varied significantly across countries. Of the selected studies, three were conducted in multidisciplinary follow-up clinics, and nine were conducted only by nurses in follow-up clinics.

### 3.2. Patient-Centered Nursing Interventions

In relation to patient-centered nursing interventions, the most frequently cited was the debriefing of ICU admission, mentioned in eleven studies [7,8,9,10,11,12,13,14,15,16,18], highlighting its broad recognition and application. Similarly, the assessment of PICS symptoms was reported in eleven studies [7,8,9,10,11,12,13,14,15,16,18], often associated with referrals to specialized care. The first nursing consultation conducted during hospitalization was emphasized in four studies [9,12,13,18], underscoring the importance of early follow-up. ICU visits after hospital discharge were also noted in four studies [9,11,13,15], as a means of helping patients process their experiences and achieve emotional closure, with some authors framing this as a form of exposure therapy.

Patient education on PICS and the promotion of well-being were highlighted in four studies [5,11,12,14], focusing on symptom awareness and strategies to enhance physical and mental health. The development of a personalized care plan was formally addressed in one study [18]. Several specific therapeutic interventions were also identified. Cognitive–behavioral therapy was mentioned in five studies [5,11,13,14,18], aiming to provide emotional support and facilitate cognitive restructuring. Group therapy, cited in two studies [16,18], was recognized for fostering social support and shared experiences. The use of a diary was described in one study [9], involving its delivery to the patient and subsequent discussion during consultations to aid in reconstructing traumatic memories. Nursing home visits were proposed in one study [18] as a strategy to ensure continuity of care post-discharge. Physical rehabilitation programs were recommended in two studies [15,16], while the use of virtual reality during follow-up consultations was mentioned in one study [10], highlighting its potential in enhancing the rehabilitation process. Additionally, one study [14] identified the distribution of informational leaflets, hospitalization photographs, and reflective documents as supportive tools.

Collectively, these interventions reflect a comprehensive and individualized approach aimed at promoting physical rehabilitation, emotional support, and patient education, thereby mitigating the physical and psychological consequences of ICU admission. The use of standardized assessment tools—such as those measuring quality of life, anxiety, sense of coherence, depression, post-traumatic stress disorder (PTSD), and caregiver burden and the ICU Memory Tool—was systematically reported in seven of the selected articles and frequently cited in the broader literature as essential for evaluating post-discharge outcomes and the effectiveness of nursing interventions.

### 3.3. Family-Centred Interventions

Regarding family-focused nursing interventions, family participation in follow-up consultations was consistently encouraged and reported in eight studies [5,7,8,11,12,14,16,18]. However, few interventions were specifically designed for family members. The provision of information to families at the time of patient discharge was emphasized in two studies [12,18], facilitating smoother transitions and recovery. The reading and sharing of patient diaries with family involvement was mentioned in one study [9], while the assessment of family well-being, coping mechanisms, and the implementation of health promotion sessions were proposed in two studies [5,18], aiming to support and empower families during the recovery process.

A study focused on the role of the family in patient recovery outlined several targeted interventions [18], including the identification of family needs and the development of a care plan, participation in group meetings to foster shared experiences, provision of emotional support and follow-up, referral and coordination with community services to enhance access to local resources, and assistance in preparing for the patient’s discharge home.

Together, these family-oriented interventions highlight the critical importance of involving family members in the recovery journey, offering them structured support and empowerment to navigate the challenges associated with ICU discharge and post-intensive care adaptation.

## 4. Discussion

The analysis of the studies in this scoping review reveals a notable variation in the structure and implementation of follow-up consultations, both in terms of the teams involved and the interventions performed. Despite this heterogeneity, nursing leadership consistently emerges as a central component, reflecting the profession’s unique positioning to provide holistic assessment, continuity of care, and psychosocial support during post-ICU recovery. This finding aligns with international evidence showing that nurses often coordinate follow-up programs and are key providers of patient-centered rehabilitation strategies [10,19,20].

### 4.1. Patient-Centered Nursing Interventions

External literature describes debriefing as a widely implemented and generally well-received strategy for helping patients make sense of their ICU experience. Studies outside the present review suggest that structured recall approaches may support the reconstruction of fragmented or delusional memories and are associated with reductions in PTSD symptoms [21,22].

In contrast, the studies included in this review present highly variable debriefing practices. Approaches range from informal conversations to diary-based discussions, and details regarding timing, facilitator preparation, or emotional-safety procedures are often sparse. This heterogeneity limits comparability and constrains interpretation regarding their intended purpose or potential influence. Some studies also note the possibility of emotional burden in the absence of supportive therapeutic structures [23].

Systematic assessment of PICS symptoms is emphasised in external guidelines as a core component of post-ICU care [24], and broader research identifies cognitive impairment as both common and frequently under-diagnosed after critical illness [25,26].

Across the included studies, assessments are common but vary considerably in the tools used and domains covered. Cognitive screening, in particular, is inconsistently reported. Such variability restricts comparison across studies and may influence how rehabilitation needs are recognised and addressed.

External qualitative studies suggest that early engagement before hospital discharge can enhance patients’ understanding of their illness and provide reassurance during recovery [27].

However, within the included studies, early consultations are reported inconsistently, and outcomes related to these interventions are rarely measured. The evidence mapped in this review does not allow conclusions regarding optimal timing or structure, indicating that early post-ICU interventions remain conceptually relevant but empirically under-explored.

ICU revisit programmes are described in external literature as potentially supporting meaning-making and the re-contextualisation of traumatic memories [27].

Among the included studies, revisits appear infrequently and differ substantially in format. Limited comparative evidence is available, and some reports highlight that revisits may be emotionally challenging for certain patients. More robust research would be required to clarify which patients might benefit and how revisits can be delivered safely.

Education is widely recognised in external literature as a critical component of patient understanding and symptom management, with some studies indicating an association between knowledge of PICS and lower psychological distress [28].

In the included studies, however, educational activities are generally informal and lack standardised materials or validated content. As a result, their scope, consistency, and potential influence cannot be clearly characterised.

While personalised, goal-directed care planning is well supported in rehabilitation research for promoting patient engagement and recovery [29], only one included study reports using this approach. Limited adoption may reflect constraints related to staffing, time, or the absence of structured frameworks in post-ICU settings.

External research suggests that structured psychological therapies may help address distress following critical illness [30].

Within the included studies, psychological support varies widely, from general supportive conversations to interventions resembling cognitive–behavioural strategies. Descriptions of training, intervention content, or underlying models are often limited, making it challenging to identify common features or intended mechanisms [31].

Group-based interventions are described in the wider literature as offering opportunities for shared meaning-making and reducing feelings of isolation among ICU survivors [32].

However, in the included studies, group sessions are rarely reported and typically described with limited detail. Reported barriers include scheduling difficulties, emotional readiness, and resource limitations. The small evidence base prevents broader interpretation.

ICU diaries are strongly supported in external literature as tools that can assist with memory reconstruction and emotional processing [22,31].

In this review, diaries were used in only one included study. Implementation details such as staff training, timing of diary delivery, and structured review processes are not consistently described. Overall, diaries appear under-utilised in the contexts represented by the included evidence.

Home visits are described in external literature as providing valuable insight into functional recovery and home-based needs [27].

Across the included studies, however, home-visit interventions are limited in number and scope. Few studies report outcomes, and resource requirements are frequently noted as barriers. Without cost-effectiveness analyses, interpretation remains constrained.

Physical rehabilitation is well supported in external literature, particularly when delivered through structured, multidisciplinary programmes [33].

In the included studies, rehabilitation often appears as standalone sessions rather than integrated pathways. These isolated approaches are described with limited detail, aligning with broader evidence that rehabilitation is typically most effective when embedded within coordinated post-ICU care.

Virtual reality (VR) is described in external research as an emerging intervention with potential for short-term anxiety relief and immersive distraction [34].

In the included studies, VR interventions remain exploratory. Sample sizes are small, protocols vary, and long-term outcomes are not reported. VR therefore appears as a promising but preliminary component of post-ICU care.

Additional tools—such as informational leaflets, hospitalisation photographs, and reflective materials—are conceptually consistent with approaches supporting memory reconstruction, including ICU diaries [35].

However, in the included studies, these tools are sparsely described and lack detail regarding implementation. Photographs, in particular, raise ethical considerations regarding consent and interpretation that are not consistently addressed. As reported, these interventions remain insufficiently characterised and inconsistently applied

### 4.2. Family-Centered Nursing Interventions

External literature highlights the value of involving family members in follow-up consultations, noting associations with improved understanding, reduced uncertainty, and greater preparedness for caregiving responsibilities [36].

Within the studies included in this review, however, family involvement is described inconsistently, and implementation procedures are seldom standardised. Few studies assess families’ needs or emotional outcomes, limiting the ability to understand how these practices are experienced or what support may be required.

Providing clear information at discharge is recognised in external studies as a key component of safe and confident caregiving, and families of ICU survivors frequently report feeling inadequately prepared for this role [27].

In the included studies, discharge-related support appears variably and is often described with limited detail regarding its content or quality. Developing structured discharge toolkits may be one potential approach to enhance consistency, although such tools were not identified in the included evidence.

External literature suggests that reading and sharing ICU diaries may help families better understand the patient’s experience and reduce uncertainty [37].

In the included studies, however, this practice is reported only once. The absence of structured guidance or facilitated review processes may limit potential benefits for families and, in some cases, could contribute to distress if sensitive content is not adequately supported.

Research outside this review identifies that assessing caregiver burden and supporting coping strategies can be associated with reductions in anxiety, depression, and strain among family members [38].

Despite this established relevance, such interventions appear infrequently in the included studies, indicating a gap between the documented burden associated with PICS-F and the limited family-centred care currently offered in follow-up contexts.

External evidence also emphasises the importance of assessing family well-being, exploring coping strategies, and providing health-promotion sessions as part of family-centred critical-care practice [38].

Only one study in this review, however, reports a structured and comprehensive approach to supporting families. This limited implementation highlights a significant gap: most follow-up services remain predominantly patient-focused, offering insufficient attention to the well-documented challenges experienced by families. Practical implications noted in the literature include conducting basic assessments of family symptoms, information needs, and contextual factors; defining referral criteria; and ensuring that follow-up services include components specifically designed for family members, such as clear information, practical guidance, and opportunities for emotional support.

In summary, the nursing interventions identified across follow-up clinics in this scoping review address several important dimensions of post-ICU recovery but remain heterogeneous, inconsistently structured, and unevenly evaluated. Interventions supported by stronger external evidence—such as ICU diaries, psychological support strategies, and structured rehabilitation—are under-represented in the included studies, while emerging approaches such as virtual reality and home-based follow-up remain exploratory and require further investigation. Together, these findings underscore the need for greater standardisation of practice, more rigorous and systematic outcome measurement, and stronger integration of family-centred approaches within post-ICU follow-up care.

### 4.3. Limitations

In this review, the search was limited to the most common databases and sought gray literature only in RCAAP, Open Dissertations via B-ON, and the references of the articles selected for title and abstract reading. A broader and more diversified search might have revealed additional relevant evidence. Also, only studies published in Portuguese, English, Spanish, and Italian were included, which may have led to the exclusion of valuable research published in other languages. However, only articles in English were ultimately found.

In accordance with JBI recommendations for scoping reviews, no assessment of methodological quality or risk of bias was performed. While this approach is consistent with the purpose of mapping existing evidence, it limits the ability to determine the robustness, reliability, and comparative strength of the included studies.

## 5. Conclusions

This scoping review suggests that nursing interventions in ICU follow-up clinics are aligned with supporting patient and family recovery after critical illness. Short-term benefits are commonly reported for interventions such as debriefing, PICS assessment, patient education, and timely referral, although evidence on long-term outcomes remains inconsistent.

Personalized care plans, multidisciplinary collaboration, and family involvement emerge as important elements of post-ICU care. Consistent use of validated outcome measures also contributes to clearer monitoring of recovery. However, substantial heterogeneity and limited standardization across follow-up clinics reduce comparability between studies. Future research should better define interventions, standardize assessments, and explore prevention strategies, PICS literacy, and community-based support to strengthen patient- and family-centered follow-up care.

## Figures and Tables

**Table 1:** Search Strategy.

Search	Expression	Results
	CINAHL Complete via EBSCO Host	
S1	TI (ICU OR “intensive care unit*” OR “critical care” OR “intensive care” OR “critical illness*” OR “critical disease”) OR AB (ICU OR “intensive care unit*” OR “critical care” OR “intensive care” OR “critical illness*” OR “critical disease”) OR MH (“critical care” OR “critical illness” OR “intensive care units”)	144,778
S2	TI (nurs* OR intervent* OR activit*) OR AB (nurs* OR intervent* OR activit*) OR MH “nursing interventions”	1,459,896
S3	TI (“PICS” OR “post-intensive care syndrome” OR “post-icu syndrome” OR “critical care syndrome” OR “critical illness syndrome” OR “PTSD” OR “post traumatic stress disorder” OR “posttraumatic stress disorder” OR “post-traumatic stress disorder”) OR AB (“PICS” OR “post-intensive care syndrome” OR “post-icu syndrome” OR “critical care syndrome” OR “critical illness syndrome” OR “PTSD” OR “post traumatic stress disorder” OR “posttraumatic stress disorder” OR “post-traumatic stress disorder”)	21,562
S4	TI (“Icu follow up” OR “follow up consultation” OR “post icu follow up” OR “post intensive care follow up” OR “post critical care follow up” OR “icu consultation” OR “intensive care consultation” OR “icu discharge” OR “intensive care discharge” OR “critical care discharge” OR “post discharge” OR “follow up clinic*” OR “critical care recovery center*” OR “icu clinic*” OR “intensive care clinic*” OR “critical care clinic*” OR “follow up” OR “follow-up”) OR AB (“Icu follow up” OR “follow up consultation” OR “post icu follow up” OR “post intensive care follow up” OR “post critical care follow up” OR “icu consultation” OR “intensive care consultation” OR “icu discharge” OR “intensive care discharge” OR “critical care discharge” OR “post discharge” OR “follow up clinic*” OR “critical care recovery center*” OR “icu clinic*” OR “intensive care clinic*” OR “critical care clinic*” OR “follow up” OR “follow-up”) OR MH (“outpatient service” OR “patient discharge” OR “post exposure follow up”)	362,504
S5 (S1 AND S2 AND S3 AND S4)	(TI (ICU OR “intensive care unit*” OR “critical care” OR “intensive care” OR “critical illness*” OR “critical disease”) OR AB (ICU OR “intensive care unit*” OR “critical care” OR “intensive care” OR “critical illness*” OR “critical disease”) OR MH (“critical care” OR “critical illness” OR “intensive care units”)) AND (TI (nurs* OR intervent* OR activit*) OR AB (nurs* OR intervent* OR activit*) OR MH “nursing interventions”) AND (TI (“PICS” OR “post-intensive care syndrome” OR “post-icu syndrome” OR “critical care syndrome” OR “critical illness syndrome” OR “PTSD” OR “post traumatic stress disorder” OR “posttraumatic stress disorder” OR “post-traumatic stress disorder”) OR AB (“PICS” OR “post-intensive care syndrome” OR “post-icu syndrome” OR “critical care syndrome” OR “critical illness syndrome” OR “PTSD” OR “post traumatic stress disorder” OR “posttraumatic stress disorder” OR “post-traumatic stress disorder”)) AND (TI (“Icu follow up” OR “follow up consultation” OR “post icu follow up” OR “post intensive care follow up” OR “post critical care follow up” OR “icu consultation” OR “intensive care consultation” OR “icu discharge” OR “intensive care discharge” OR “critical care discharge” OR “post discharge” OR “follow up clinic*” OR “critical care recovery center*” OR “icu clinic*” OR “intensive care clinic*” OR “critical care clinic*” OR “follow up” OR “follow-up”) OR AB (“Icu follow up” OR “follow up consultation” OR “post icu follow up” OR “post intensive care follow up” OR “post critical care follow up” OR “icu consultation” OR “intensive care consultation” OR “icu discharge” OR “intensive care discharge” OR “critical care discharge” OR “post discharge” OR “follow up clinic*” OR “critical care recovery center*” OR “icu clinic*” OR “intensive care clinic*” OR “critical care clinic*” OR “follow up” OR “follow-up”) OR MH (“outpatient service” OR “patient discharge” OR “post exposure follow up”))	139
S6	SubjectAge:—all adult	54
	PubMed	
#1	(((ICU[Title/Abstract] OR “intensive care unit*”[Title/Abstract] OR “critical care”[Title/Abstract] OR “intensive care”[Title/Abstract] OR “critical illness*”[Title/Abstract] OR “critical disease”[Title/Abstract]) OR (“critical care”[MeSH Terms])) OR (“critical illness”[MeSH Terms])) OR (“intensive care units”[MeSH Terms])	340,946
#2	nurs*[Title/Abstract] OR intervent*[Title/Abstract] OR activit*[Title/Abstract]	5,562,066
#3	(“PICS”[Title/Abstract] OR “post-intensive care syndrome”[Title/Abstract] OR “post-icu syndrome”[Title/Abstract] OR “critical care syndrome”[Title/Abstract] OR “critical illness syndrome”[Title/Abstract] OR “PTSD”[Title/Abstract] OR “post traumatic stress disorder”[Title/Abstract] OR “posttraumatic stress disorder”[Title/Abstract] OR “post-traumatic stress disorder”[Title/Abstract]) OR (“Stress Disorders, Post-Traumatic”[MeSH Terms])	63,777
#4	((((“Icu follow up”[Title/Abstract] OR “follow up consultation”[Title/Abstract] OR “post icu follow up”[Title/Abstract] OR “post intensive care follow up”[Title/Abstract] OR “post critical care follow up”[Title/Abstract] OR “icu consultation”[Title/Abstract] OR “intensive care consultation”[Title/Abstract] OR “icu discharge”[Title/Abstract] OR “intensive care discharge”[Title/Abstract] OR “critical care discharge”[Title/Abstract] OR “post discharge”[Title/Abstract] OR “follow up clinic*”[Title/Abstract] OR “critical care recovery center*”[Title/Abstract] OR “icu clinic*”[Title/Abstract] OR “intensive care clinic*”[Title/Abstract] OR “critical care clinic*”[Title/Abstract] OR “follow up”[Title/Abstract] OR “follow-up”[Title/Abstract]) OR (“patient discharge”[MeSH Terms])) OR (aftercare[MeSH Terms])) OR (“ambulatory care”[MeSH Terms])) OR (“Ambulatory Care Facilities”[MeSH Terms])	1,634,254
#5	((((((ICU[Title/Abstract] OR “intensive care unit*”[Title/Abstract] OR “critical care”[Title/Abstract] OR “intensive care”[Title/Abstract] OR “critical illness*”[Title/Abstract] OR “critical disease”[Title/Abstract]) OR (“critical care”[MeSH Terms])) OR (“critical illness”[MeSH Terms])) OR (“intensive care units”[MeSH Terms])) AND (nurs*[Title/Abstract] OR intervent*[Title/Abstract] OR activit*[Title/Abstract])) AND ((“PICS”[Title/Abstract] OR “post-intensive care syndrome”[Title/Abstract] OR “post-icu syndrome”[Title/Abstract] OR “critical care syndrome”[Title/Abstract] OR “critical illness syndrome”[Title/Abstract] OR “PTSD”[Title/Abstract] OR “post traumatic stress disorder”[Title/Abstract] OR “posttraumatic stress disorder”[Title/Abstract] OR “post-traumatic stress disorder”[Title/Abstract]) OR (“Stress Disorders, Post-Traumatic”[MeSH Terms]))) AND (((((“Icu follow up”[Title/Abstract] OR “follow up consultation”[Title/Abstract] OR “post icu follow up”[Title/Abstract] OR “post intensive care follow up”[Title/Abstract] OR “post critical care follow up”[Title/Abstract] OR “icu consultation”[Title/Abstract] OR “intensive care consultation”[Title/Abstract] OR “icu discharge”[Title/Abstract] OR “intensive care discharge”[Title/Abstract] OR “critical care discharge”[Title/Abstract] OR “post discharge”[Title/Abstract] OR “follow up clinic*”[Title/Abstract] OR “critical care recovery center*”[Title/Abstract] OR “icu clinic*”[Title/Abstract] OR “intensive care clinic*”[Title/Abstract] OR “critical care clinic*”[Title/Abstract] OR “follow up”[Title/Abstract] OR “follow-up”[Title/Abstract]) OR (“patient discharge”[MeSH Terms])) OR (aftercare[MeSH Terms])) OR (“ambulatory care”[MeSH Terms])) OR (“Ambulatory Care Facilities”[MeSH Terms]))	378
#6	Filters: Adult: 19+ years	171
	Web of Science	
1	TS=(ICU OR “intensive care unit*” OR “critical care” OR “intensive care” OR “critical illness*” OR “critical disease”) AND TS=(nurs* OR intervent*) AND TS=(“PICS” OR “post-intensive care syndrome” OR “post-icu syndrome” OR “critical care syndrome” OR “critical illness syndrome” OR “PTSD” OR “post traumatic stress disorder” OR “posttraumatic stress disorder” OR “post-traumatic stress disorder”) AND TS=(“Icu follow up” OR “follow up consultation” OR “post icu follow up” OR “post intensive care follow up” OR “post critical care follow up” OR “icu consultation” OR “intensive care consultation” OR “icu discharge” OR “intensive care discharge” OR “critical care discharge” OR “post discharge” OR “follow up clinic*” OR “critical care recovery center*” OR “icu clinic*” OR “intensive care clinic*” OR “critical care clinic*” OR “follow up” OR “follow-up”) AND TS=(adult* OR “young adult*” OR “middle aged” OR aged OR elderly)	165
	RCAAP e Open Dissertations via B-ON	
S1	TI (ICU OR “intensive care unit*” OR “critical care” OR “intensive care” OR “critical illness*” OR “critical disease”) OR AB (ICU OR “intensive care unit*” OR “critical care” OR “intensive care” OR “critical illness*” OR “critical disease”) OR SU (ICU OR “intensive care unit*” OR “critical care” OR “intensive care” OR “critical illness*” OR “critical disease”)	1,568,760
S2	TI (nurs* OR intervent* OR activit*) OR AB (nurs* OR intervent* OR activit*) OR SU (nurs* OR intervent* OR activit*)	26,573,420
S3	TI (“PICS” OR “post-intensive care syndrome” OR “post-icu syndrome” OR “critical care syndrome” OR “critical illness syndrome” OR “PTSD” OR “post traumatic stress disorder” OR “posttraumatic stress disorder” OR “post-traumatic stress disorder”) OR AB (“PICS” OR “post-intensive care syndrome” OR “post-icu syndrome” OR “critical care syndrome” OR “critical illness syndrome” OR “PTSD” OR “post traumatic stress disorder” OR “posttraumatic stress disorder” OR “post-traumatic stress disorder”) OR SU (“PICS” OR “post-intensive care syndrome” OR “post-icu syndrome” OR “critical care syndrome” OR “critical illness syndrome” OR “PTSD” OR “post traumatic stress disorder” OR “posttraumatic stress disorder” OR “post-traumatic stress disorder”)	369,142
S4	TI (“Icu follow up” OR “follow up consultation” OR “post icu follow up” OR “post intensive care follow up” OR “post critical care follow up” OR “icu consultation” OR “intensive care consultation” OR “icu discharge” OR “intensive care discharge” OR “critical care discharge” OR “post discharge” OR “follow up clinic*” OR “critical care recovery center*” OR “icu clinic*” OR “intensive care clinic*” OR “critical care clinic*” OR “follow up” OR “follow-up”) OR AB (“Icu follow up” OR “follow up consultation” OR “post icu follow up” OR “post intensive care follow up” OR “post critical care follow up” OR “icu consultation” OR “intensive care consultation” OR “icu discharge” OR “intensive care discharge” OR “critical care discharge” OR “post discharge” OR “follow up clinic*” OR “critical care recovery center*” OR “icu clinic*” OR “intensive care clinic*” OR “critical care clinic*” OR “follow up” OR “follow-up”) OR SU (“Icu follow up” OR “follow up consultation” OR “post icu follow up” OR “post intensive care follow up” OR “post critical care follow up” OR “icu consultation” OR “intensive care consultation” OR “icu discharge” OR “intensive care discharge” OR “critical care discharge” OR “post discharge” OR “follow up clinic*” OR “critical care recovery center*” OR “icu clinic*” OR “intensive care clinic*” OR “critical care clinic*” OR “follow up” OR “follow-up”)	2,499,960
S5	TI (adult OR “young adult” OR “middle age” OR aged OR elderly) OR AB (adult OR “young adult” OR “middle age” OR aged OR elderly) OR SU (adult OR “young adult” OR “middle age” OR aged OR elderly)	17,589,905
S6	S1 AND S2 AND S3 AND S4 AND S5	274
S7	Limitadores—Restringir por: Fornecedor de Conteúdos: Open Dissertations, RCAAP	4

**Table 2:** Data extraction table.

Authors/Year/Country	Study Type	Objective	Population	Patient Interventions	Patient Outcomes	Family Interventions	Family Outcomes
Henderson et al. (2021), Scotland [7]	RCT	Assess long-term outcomes and feasibility of Inspire program	27 patients; 23 caregivers	Multidisciplinary rehab; ICU debriefing; Support groups; Psychosocial assessments	Improved QoL; Reduced anxiety/depression	Family meetings	Reduced anxiety/depression
Jensen et al. (2015), England [8]	Systematic review & Meta-analysis	Evaluate follow-up consultations vs standard care	855 ICU survivors	ICU debriefing; QoL and mental health assessments	No significant impact; Possible PTSD reduction at 3–6 months	None reported	Not applicable
Petersson et al. (2015), Sweden [9]	Descriptive Comparative	Explore ICU diaries and memory recall	96 patients (52 intervention, 44 control)	ICU diary; Ward visit; Follow-up consultations; Memory/PTSD scales	Diaries aided understanding; No difference in PTSD scores	Diary sharing; Consultation attendance	Diaries perceived as valuable
Vlake et al. (2022), Netherlands [10]	RCT	Assess ICU-VR impact on mental health and satisfaction	89 patients (45 intervention, 44 control)	ICU-VR video; Psychological assessments; Follow-up consultations	Improved satisfaction; No change in recovery/QoL	None reported	Not applicable
Hanifa et al. (2018), Germany [11]	Ethnographic Study	Explore follow-up consultation experiences	10 patients	ICU debriefing; Emotional support; ICU visit; Referrals	Better understanding of PICS; ICU visit seen as therapeutic	Family invited to attend	Not reported
Jónasdóttir et al. (2018), Iceland [12]	Quasi-experimental	Compare structured nurse-led vs standard follow-up	168 patients (83 intervention, 85 control)	Ward visits; Phone interviews; ICU debriefing; Psychological scales	Higher PTSD/anxiety in intervention group	Info at discharge; ICU visit invitation	Not reported
Valsø et al. (2020), Norway [13]	RCT	Assess nurse-led consultations PTSD and coherence	523 patients (111 intervention, 113 control)	3 consultations; CBT and narrative methods; ICU visit	PTSD reduced in both groups; Low coherence linked to higher PTSD	None reported	Not applicable
Jensen et al. (2016), Denmark [14]	RCT	Evaluate post-ICU recovery program vs standard care	386 patients	3 consultations; Debriefing; Reflection sheets; Health education	Reduced anxiety/depression; No change in PTSD/QoL	Family attended first consultation	Not reported
Cuthbertson et al. (2009), England [15]	RCT	Test effectiveness and cost-efficiency of nurse-led follow-up	286 patients	Rehab manual; Consultations at 3 & 9 months; ICU visit; Psychological scales	No evidence of effectiveness or cost-efficiency	Not reported	Not applicable
McPeake et al. (2017), Scotland [16]	Cohort Study	Evaluate Inspire program on QoL and self-efficacy	49 patients	5-week rehab; Individual/group sessions; ICU debriefing	Improved QoL and self-efficacy; Higher return to work	Caregivers could attend sessions	Not reported
Ågren et al. (2019), Sweden [17]	RCT	Assess nurse-led family intervention on well-being	45 families (17 recruited)	Standard follow-up + 3 health promotion sessions	Not specified	3 nurse-led sessions; Problem-solving; Summary letter	Improved family function; Reduced stress; No change in hope
Daly et al. (2005), USA [18]	RCT	Test patient management program on readmissions and cost	334 patients (231 intervention, 103 control)	Pre-discharge planning; Home visits; Monitoring; Referrals	35% fewer readmission days; Cost savings	Pre-discharge planning; Emotional support; Community coordination	Qualitative benefits reported

**Table 3:** Nursing interventions summary.

Nursing Interventions for Patients	Nursing Interventions for Families
- Debriefing of hospitalization [7,8,9,10,11,12,13,14,15,16,18] - Assessment of PICS symptoms [7,8,9,10,11,12,13,14,15,16,18] - First nursing consultation in the ward [9,12,13,18] - ICU visits after hospital discharge [9,11,13,15] - Education on PICS and promotion of well-being [5,11,12,14] - Development of a personalized care plan [18] - Specific therapies [5,11,13,14,16,18] - Nursing home visits [18] - Physical rehabilitation [15,16] - Virtual Reality [10] - Provision of informational leaflets, hospitalization photographs, and reflective documents [14]	- Participation in follow-up consultations [5,7,8,11,12,14,16,18] - Provision of information to families at the time of patient discharge [12,18] - Reading and sharing of patient diaries with family [9] - Assessment of family well-being, coping mechanisms, and the implementation of health promotion sessions [5,18]

## Data Availability

All data generated or analyzed during this scoping review are included in this published article and its Supplementary Information Files. No primary datasets were created or collected for this study, as it is based exclusively on previously published literature.

## Supplementary Materials

The following supporting information can be downloaded at: https://www.mdpi.com/article/10.3390/nursrep16020055/s1, PRISMA 2020 Checklist. Reference [6] is cited in the Supplementary Materials.

## Public Involvement Statement

No patients or members of the public were involved in the design, conduct, or reporting of this scoping review. The study relied exclusively on previously published literature.

## Guidelines and Standards Statement

This scoping review was conducted in accordance with the Joanna Briggs Institute (JBI) methodology for scoping reviews and is reported following the Preferred Reporting Items for Systematic Reviews and Meta-Analyses extension for Scoping Reviews (PRISMA-ScR) checklist.
